# PSO Based PI Controller Design for a Solar Charger System

**DOI:** 10.1155/2013/815280

**Published:** 2013-05-13

**Authors:** Her-Terng Yau, Chih-Jer Lin, Qin-Cheng Liang

**Affiliations:** ^1^Department of Electrical Engineering, National Chin-Yi University of Technology, Taichung 41170, Taiwan; ^2^Graduate Institute of Automation and Technology, National Taipei University of Technology, Taipei 10608, Taiwan

## Abstract

Due to global energy crisis and severe environmental pollution, the photovoltaic (PV) system has become one of the most important renewable energy sources. Many previous studies on solar charger integrated system only focus on load charge control or switching Maximum Power Point Tracking (MPPT) and charge control modes. This study used two-stage system, which allows the overall portable solar energy charging system to implement MPPT and optimal charge control of Li-ion battery simultaneously. First, this study designs a DC/DC boost converter of solar power generation, which uses variable step size incremental conductance method (VSINC) to enable the solar cell to track the maximum power point at any time. The voltage was exported from the DC/DC boost converter to the DC/DC buck converter, so that the voltage dropped to proper voltage for charging the battery. The charging system uses constant current/constant voltage (CC/CV) method to charge the lithium battery. In order to obtain the optimum PI charge controller parameters, this study used intelligent algorithm to determine the optimum parameters. According to the simulation and experimental results, the control parameters resulted from PSO have better performance than genetic algorithms (GAs).

## 1. Introduction

Effective utilization of solar energy is an important research subject of photovoltaic (PV) system at present, and using the electric energy generated by solar cell is the key. The electric energy generated by solar cell is very likely to be influenced by environmental factors, such as irradiance conditions, solar angle, temperature variation, and instability of output power or shadow, which would result in power loss of solar cell [1]. 

Therefore, there must be an electric energy controller with good robustness and stability to ensure the effective utilization of solar energy, in order for the overall solar power supply system to generate maximum electric energy efficiently and maintain the balanced and stable control of system [2]. 

Recharging of secondary battery efficiently is very important. The chargers only charged batteries in the past, and the charging method and efficiency were not designed well, so that the excessive charging time or frequency often damaged batteries and reduced the lifetime. In order to improve the life or charging effect of secondary battery, there must be a good charge control design for battery, so that the battery life can be prolonged and the charge efficiency can be increased [3–5]. 

Many studies of the charger of PV system use single-buck converter to discuss the overall MPPT and charge control system [6]. The defect in using single-buck converter to control the overall system is that one converter only controls one system; in order to achieve the MPPT of solar energy and to control the lithium battery charging, there must be a switching mechanism to identify the control method selected by the system. This single-buck converter system is unable to implement MPPT and charge control simultaneously. When this single-stage design is applied to MPPT, the lithium battery load has not controlled the voltage and current and the battery life may be reduced.

This study designed a portable solar energy lithium battery charger, on the principle of two-stage system. The first stage uses a DC/DC boost converter, which adopts the variable step size incremental conductance (VSINC) method to control the solar power system working at the maximum power point [7–9]. The second stage uses a DC/DC buck converter [10], which controls the charging system by battery voltage and current feedback for the lithium battery, so as to make the system attain constant voltage, current, and voltage charge. The PI controller based on optimal algorithm is designed to achieve the charge control of second stage [11, 12]. This two-stage system can achieve simultaneous MPPT of solar energy and lithium charge control. The two-stage design not only controls the two-stage system but also there is a control system when the battery is being charged, thus prolonging the charge cycle of lithium battery. This study used Simulink and Simpower of MATLAB simulation software for simulation analysis of system. In terms of portable hardware, the embedded PIC18F8720 microcontrol chip developed by Microchip was used to compose the MPPT algorithm and battery charge controller. The experimental results were compared with simulation result to prove the effect of the proposed control theory.

## 2. Solar Cell Characteristics

Solar cell is mainly made of PV wafers, converts the light energy of solar irradiation into voltage and current directly for load, and conducts electricity without electrolytic effect. The electric energy is obtained from the PN interface of semiconductor directly; therefore, the solar cell is also known as PV cell [1, 2].

The mathematical model of solar cells in series or parallel connection can be simply expressed as

(1) $$\begin{array}{c} I_{\text{PV}}=I_{\text{ph}}-I_{\mathrm{sat}}\left(\exp\frac{q}{BkT}V_{\text{PV}}-1\right), \end{array}$$

where *I*_PV_ is output current of solar cell (A); *V*_PV_ is output voltage of solar cell (V); *I*_sat⁡_ is reverse saturation current of solar cell; *I*_ph_ is current output of solar cell; *q* is quantity of electronic charge; *k* is boltzmann constant; *B* is ideal factor of solar cell; and *T* is solar cell surface temperature.

**Figure 1:**
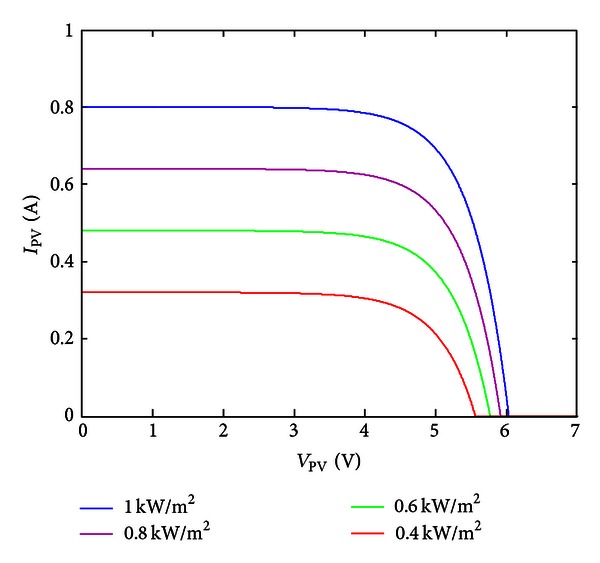
Current-voltage characteristic curve diagram of solar cell under varying irradiance.

**Figure 2:**
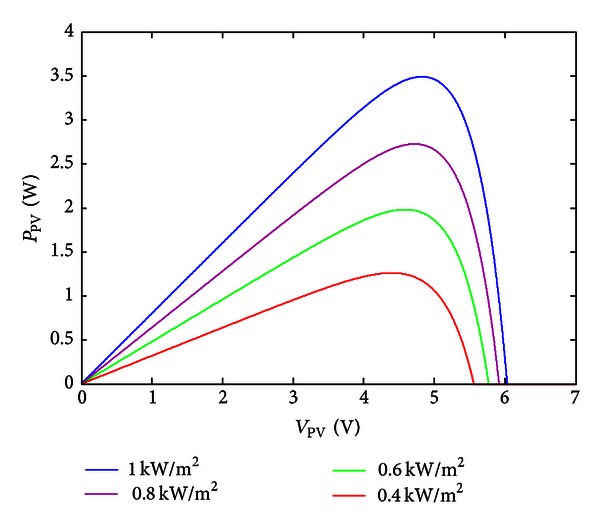
Power-voltage curve diagram of solar cell under varying irradiance.

The power-voltage and current-voltage curves of mathematical model corresponding to solar cell in different illuminations are shown in Figures 1 and 2.

## 3. Maximum Power Point System Design

**Figure 3:**
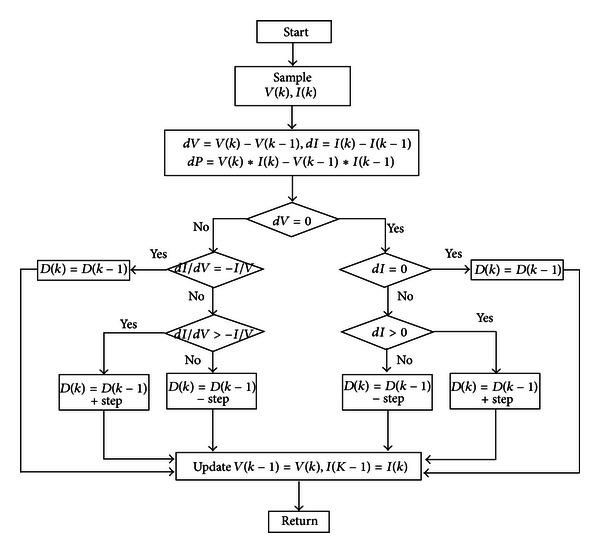
Flow chart of VSINC.

In application, the MPPT algorithm is between the solar cell and DC/DC boost converter. The solar cell output uses the duty cycle of direct control power converter to reduce the complexity of system [13]. The process of MPPT algorithm of this modified VSINC method is shown in Figure 3. The step size of MPPT algorithm of traditional incremental conductance is fixed. If large step size control is adopted, the solar cell can track the maximum power point rapidly, but the large step size tracking can cause much oscillation in relatively steady state. This result will reduce the output efficiency. On the contrary, it tracks the maximum power point slowly and the oscillation is very small in steady state. Therefore, the step size fixed incremental conductance method must make choices between transient and steady state. This study used VSINC [14], which can track the maximum power point rapidly in transient. The steady-state oscillation is very small, so that the tracking time can be shortened, and it has excellent efficiency, as compared with traditional incremental conductance method. 

The duty cycle of converter is modulated automatically. In the system of duty cycle of solar cell output power direct control converter, *V*(*t*) and *I*(*t*) are the voltage and current of solar cell output at time *t*, and *D*(*t*) and step are the variation of duty cycle and step size. The design of variable step size reduces the problems of slow tracking of small step size and large steady-state oscillation of large step size of traditional incremental conductance method, expressed as the following equation [14]:

(2) $$\begin{array}{c} D\left(t\right)=D\left(t-1\right)\pm \text{step}, \end{array}$$

where step is the variable step size variation of duty cycle variation, and this variation can be expressed as

(3) $$\begin{array}{c} \text{step}=K\times \left|\frac{dP}{dV}\right|. \end{array}$$

|*dP*|/|*dV*| is identification factor of step change of VSINC, and the differentials of *P* and *V* are determined from the power-voltage output characteristic curve of solar cell. The step size is identified, and *K* is the scaling factor of duty cycle. Therefore, (2) can be changed to the following equation:

(4) D(t)=D(t−1)±K×|dPdV|=D(t−1)±K×|P(t)−P(t−1)V(t)−V(t−1)|.

Here the performance of MPPT depends on the scaling factor *K* and the tuning of this parameter depends on the system efficiency. The operation process of the algorithm used in this paper is described below as follows.

(1) The step size uses Δ*D*_max⁡_ as initial tracking condition, so that the system tracks the maximum power point rapidly.
(2) The variable step size scaling condition of this Δ*D*_max⁡_ is evaluated in steady state; here |*dP* | /|*dV*| is in the minimum value of maximum power point.
(3) The variable step size feeds back update condition to ensure the convergence; here the variable step size variation must meet the following condition [14]:
  (5) $$\begin{array}{c} \Delta D_{\max}\left(t\right)+K\times \left|\frac{dP}{dV}\right|<\Delta D_{\max}\left(t-1\right), \end{array}$$
so the condition of scaling factor is
  (6) $$\begin{array}{c} K<\frac{\Delta D_{\max}\left(t-1\right)}{\left[\left|dP/dV\right|+\Delta D_{\max}\left(t\right)\right]}. \end{array}$$

**Figure 4:**
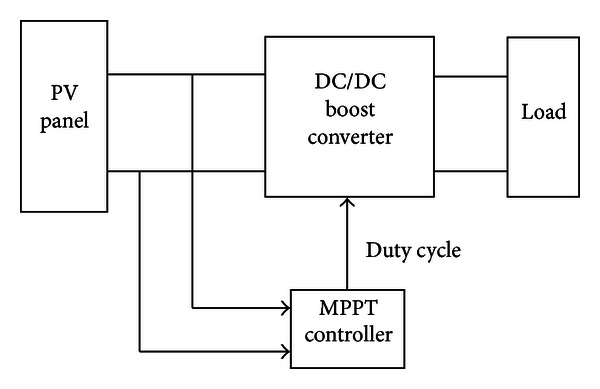
MPPT control system of solar cell.

The solar energy MPPT controller in this paper can be implemented by a DC/DC boost converter, and the system is shown in Figure 4. The MPPT controller is the VSINC.

## 4. Design of Charging System

The constant current/constant voltage method is the most practical at present. The charging process of this method is consisted of two stages. First, the lithium battery is charged with constant current, so as to shorten the charging time. When the battery voltage reaches the required set value, it is charged with constant voltage. The charging current decreases gradually as the time extends, and the charger is cut off until the charging current decreases to about 0 [15–17]. 

The charging method used in this study connects a DC/DC buck converter to the preceding stage output, so that the voltage and current of preceding stage maximum power control the constant current/constant voltage charge-up method for lithium battery. The constant current/constant voltage architecture is that the fed back output voltage uses a PI controller to control the duty cycle for charging. Since the PI controller can suppress high-frequency noise to improve the system or eliminate steady-state error, the battery output achieves stable constant current/constant voltage control. 

**Figure 5:**
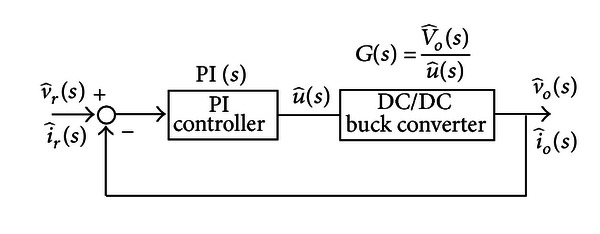
Linearized closed loop control system.

Take constant voltage as an example, the charging system uses state space averaging method to analyze the PI controller and DC/DC buck converter. This method can linearize the nonlinear system equation of DC/DC buck converter, so as to establish the transfer function of integrated charging system.  Figure 5 shows the linearized closed loop control system of charging system.

**Figure 6:**
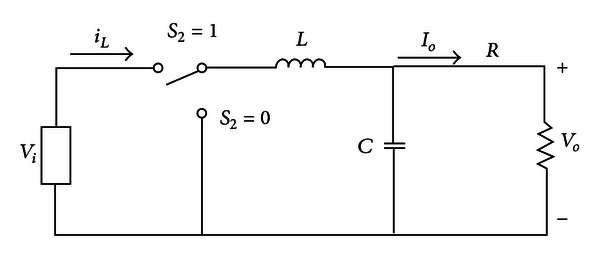
DC/DC buck converter.

Figure 6 shows the on-off equivalent circuit of DC/DC buck converter, and the input and output transfer functions are deduced from this system:

(7) $$\begin{array}{c} G\left(s\right)=\frac{{\hat{V}}_{o}\left(s\right)}{\hat{u}\left(s\right)}=\frac{sC+1}{LC\left(s^{2}+\left(1/RC\right)s+\left(1/LC\right)\right)}. \end{array}$$

Equation (7) can be expressed as

(8) $$\begin{array}{c} G\left(s\right)=\frac{{\hat{i}}_{o}\left(s\right)}{\hat{u}\left(s\right)}=\frac{sC+1}{LCR\left(s^{2}+\left(1/RC\right)s+\left(1/LC\right)\right)}\times V_{i}, \end{array}$$

where *i*_*o*_ is the output current.

The transfer function of DC/DC buck converter (7) is combined with PI controller to deduce the loop circuit transfer function *T*(*s*) of overall constant voltage charging system

(9) T(s)=v^o(s)v^r(s)=PI(s)G(s)1+PI(s)G(s)=kPLs2+(kPLC+kIL)s+kILC×(s3+(1RC+kPL)s2  +(kPLC+kIL+1LC)+kILC)−1.

This transfer function *T*(*s*) is substituted in the RLC parameter value of this system to obtain

(10) T(s)=a1kPs2+(5×107kP+a1kI)s+5×107kI×(s3+(5×107+a1kP)s2  +(5×107kP+a1kI+5×107)s+5×107kI)−1,

where *a*_1_ = 250000.

This system is loop circuit transfer function, where *A*(*s*) is the characteristic equation as (10)

(11) A(s)=s3+(5×107+a1kp)s2+(5×107kp+a1ki+5×107)s+5×107kI.

The result of the characteristic equation calculated by Routh table is that if *k*_*P*_ and *k*_*I*_ are greater than zero, the poles of this system are in the left half plane of s plane, meaning that the PI controller can control the stability of this system. 

The transfer function of DC/DC buck converter (12) is combined with PI controller to deduce the loop circuit transfer function *T*(*s*) of overall constant-current charging system:

(12) T(s)=i^o(s)i^r(s)=PI(s)G(s)1+PI(s)G(s)=kPViLRs2+(kPViLCR+kIViLR)s+kIViLCR=(s3+(1CR+kPViLR)s2   +(kPViLCR+kIViLR+1LC)s+kIViLCR)−1,

where *V*_*i*_ is the input voltage of DC/DC buck converter; this transfer function *T*(*s*) is substituted in the RLC parameter value of this system to obtain

(13) $$\begin{array}{c} T\left(s\right)=\frac{a_{2}k_{P}s^{2}+\left(10^{8}k_{P}+a_{2}k_{I}\right)s+10^{8}k_{I}}{s^{3}+\left(10^{8}+a_{2}k_{P}\right)s^{2}+\left(10^{8}k_{P}+a_{2}k_{I}+10^{8}\right)+10^{8}k_{I}}, \end{array}$$

where *a*_2_ = 500000.

This system is loop circuit transfer function, where *A*(*s*) is the characteristic equation as (13)

(14) A(s)=s3+(108+a2kP)s2+(108kP+a2kI+108)+108kI.

The result of the characteristic equation calculated by Routh table is that if *k*_*P*_ and *k*_*I*_ are greater than 0, the poles of this system are in the left half plane of s plane, meaning that the PI controller can control the stability of this system. 

**Figure 7:**
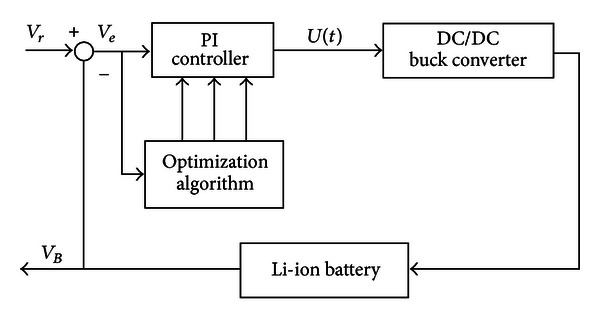
Optimization algorithm system of DC/DC buck converter.

The PI controller used in the second-stage charging system certainly can make the system achieve steady-state response under its control; this study focused on selecting the *k*_*P*_ and *k*_*I*_ values of PI controller to make the charging system implementing optimal constant voltage and constant current/constant voltage charge. Therefore, this study used the Genetic Algorithm (GA) and Particle Swarm Optimization (PSO) of optimization algorithm to search for and compare *k*_*P*_ and *k*_*I*_ values [18–21]. The system structure block is shown in Figure 7.

## 5. Simulation Result of Portable Solar Energy Charging System 

**Figure 8:**
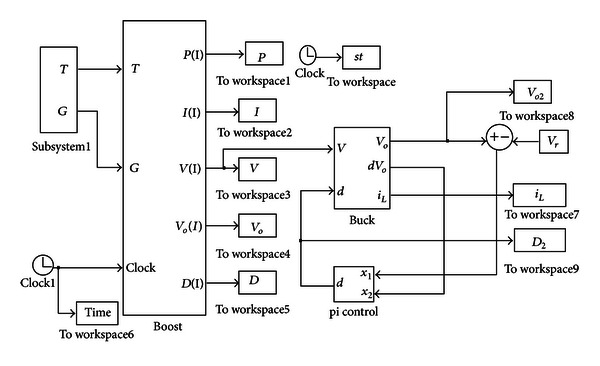
Solar charger simulation system.

This study used Simulink of MATLAB simulation software to construct the mathematical model of overall system, including the boost converter of the first stage and the buck converter of the second stage. The duty cycle control input of the first stage boost converter uses MPPT algorithm for input voltage stabilization and output boost control of the boost converter connected to the solar cell. The duty cycle control input of the second-stage buck converter uses battery fed back current and voltage for charge control, so that the lithium battery can be charged stably.  Figure 8 shows the overall simulation system of this study. 

### 5.1. MPPT Simulation Result

The MPPT algorithm simulation in this study compares the VSINC method with the general incremental conductance method (large step size and small step size), simulated in standard test conditions (1 KW/m^2^, A.M. 1.5, 25°C) and varying irradiance condition (1 KW/m^2^ and 800 W/m^2^). The tracked maximum power of the solar cell used in this study is 3.5 W when the irradiance is 1 KW/m^2^, and the tracked maximum power is 2.7 W when the irradiance is 800 W/m^2^.

**Figure 9:**
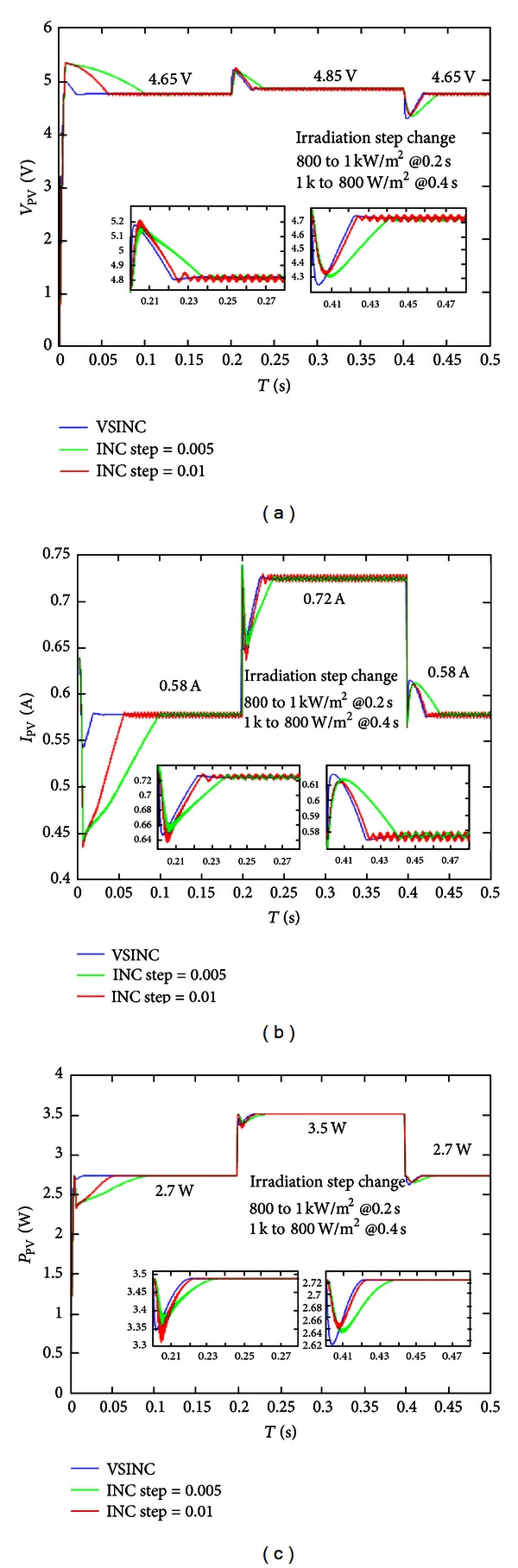
Comparison of best power point: (a) voltage, (b) current, and (c) power in changed irradiance condition (800 W/m^2^-1 KW/m^2^-800 W/m^2^).

Figure 9 compares the voltage, current, and power of fixed step size incremental conductance method when the irradiance is changed from 800 W/m^2^ to 1 KW/m^2^ and to 800 W/m^2^ with that of the VSINC method. As seen, the VSINC method used in this study has better efficiency than the traditional fixed step size incremental conductance method. The maximum power point tracking time is much shorter when the PV system is started up, and the maximum power point can be tracked faster than traditional fixed step size incremental conductance method when the irradiance is changed. According to the comparison of voltage and current, whether the irradiance is increased or decreased, the steady-state oscillation of tracking maximum power of PV system by the VSINC method is less than that by traditional fixed step size incremental conductance method.

### 5.2. Simulation Result of Charge Control

This charging system uses GA and PSO to search for *k*_*P*_ and *k*_*I*_ values of the PI controller in the charging system and then uses constant voltage and constant current/constant voltage method to simulate the charging system. The lithium battery used in this paper is 3.7 V, 3000 mA/H, and the battery saturation voltage is 4.2 V. 

The simulation system uses optimization evolution method to find the *k*_*P*_ and *k*_*I*_ values of feedback PI controller, and then they are substituted in the system for comparison. The fitness function IAE design of this study will take absolute value integral from input-output error and uses the maximum value of each calculation process as the adaptive value. This fitness function is expressed as follows:

(15) $$\begin{array}{c} \text{IAE}=\int_{0}^{\infty }\left|e\left(t\right)\right|dt, \end{array}$$

where *e*(*t*) is the current under ideal control, subtracting error values of feedback output voltage, and output current values from voltage.

The algorithm adjusts the PI controller according to the fitness function of (15) so as to converge the steady-state error to 0. The voltage and current of the second-stage charging system can be controlled stably, and the system implements constant voltage and constant current/constant voltage charge. 

**Figure 10:**
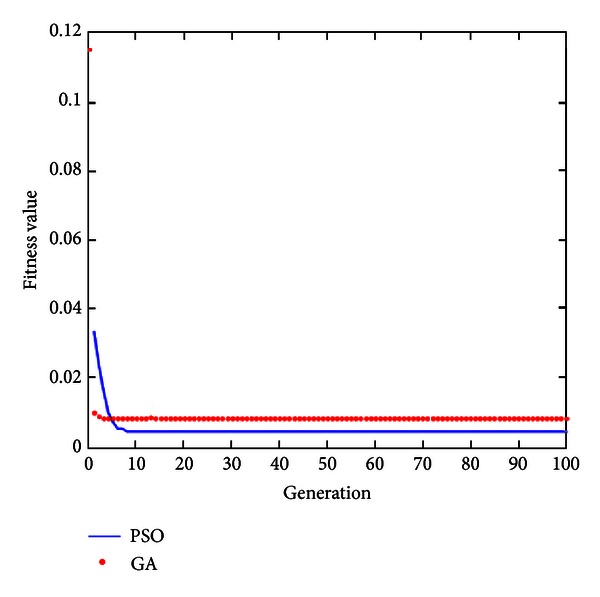
IAE curve diagram of GA and PSO.

This study used a feedback voltage charging system to control the constant voltage charge for saturation voltage 4.2 V of lithium battery and used feedback current to control the constant current at 1 A to charge the battery. The constant voltage charge-up method and constant current/constant voltage charge-up method were discussed and compared.  Figure 10 is the IAE convergence curve diagram of optimization algorithm for constant voltage charge. As seen, the GA can search for the optimal solution rapidly, and although the convergence rate of PSO is lower than that of GA, the convergence value of PSO's optimal solution is better than GA.

**Figure 11:**
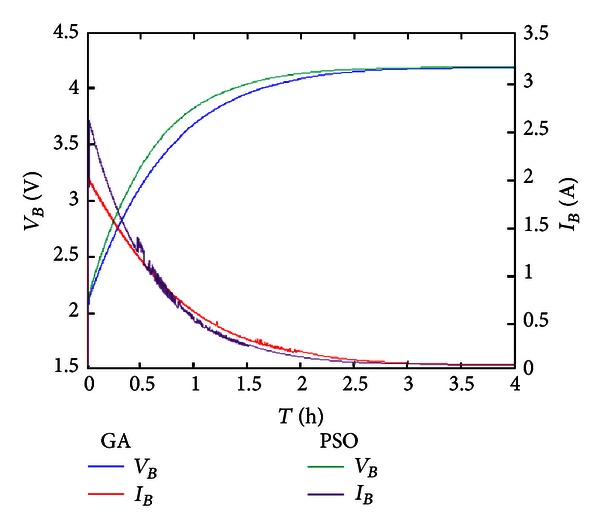
Constant voltage charge using optimization algorithm.

Figure 11 shows the simulation result of constant voltage charge using PI controller. The *k*_*P*_ and *k*_*I*_ values of PI controller are adjusted by GA and PSO, respectively. As seen, whether we use PSO or GA, the adjusted *k*_*P*_ and *k*_*I*_ values of PI controller can stabilize the power supplied from the first-stage converter, so as to supply electric energy soundly to charge the second-stage charging system effectively. Moreover; the PSO adjusted PI controller is better than GA; the PSO adjusted constant voltage PI controller can use the electric energy generated by the first-stage PV system effectively and charges the battery faster than the GA adjusted constant voltage PI controller.

**Figure 12:**
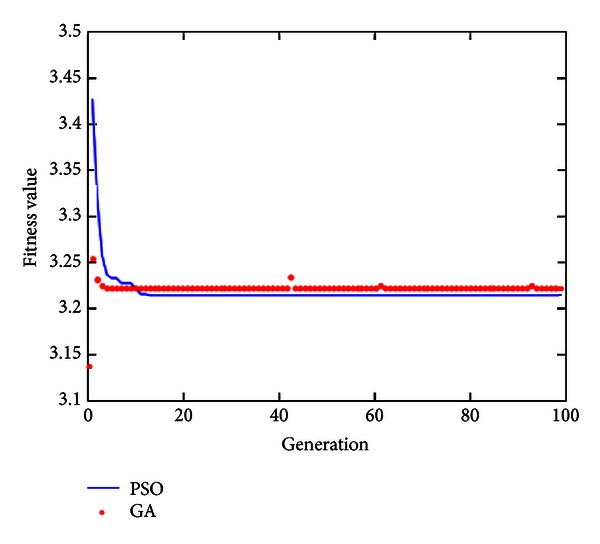
IAE curve diagram of GA and PSO.

Figure 12 shows the IAE convergence curve of optimization algorithm for constant current/constant voltage charge. As seen, in constant-current charge, the convergence rate of GA is similar to that of PSO. In this constant-current state, the convergence value of PSO is better than that of GA.

**Figure 13:**
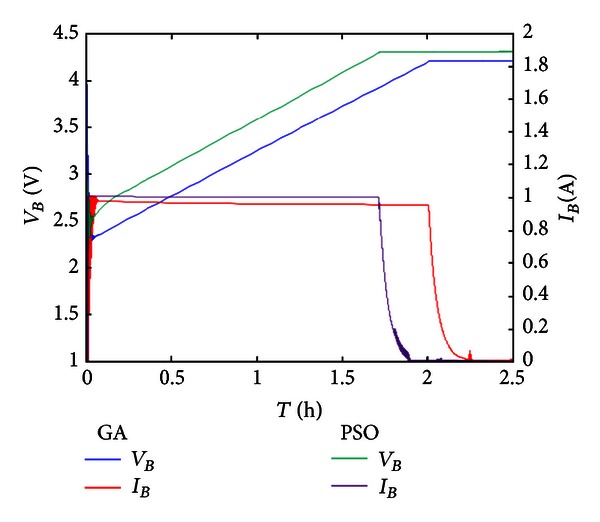
Constant voltage charge using optimization algorithm.

Figure 13 shows the simulation result of constant current/constant voltage charge using PI controller. The constant current/constant voltage charge in this study switches the battery to constant-current charge to obtain 4.18 V charging voltage and then switches to constant voltage charge. The charging system uses GA and PSO to adjust the *k*_*P*_ and *k*_*I*_ values of PI controller, respectively. In constant current/constant voltage charging, whether we use PSO or GA, the *k*_*P*_ and *k*_*I*_ values of the adjusted PI controller can make the second-stage charging system charge effectively. 

The GA adjusted PI controller has not implemented constant-current charge of system effectively, so that the charging time is prolonged, whereas the PSO adjusted PI controller can implement accurate constant-current charge of the secondary charging system. In addition, in the second-stage constant voltage charge, any optimization algorithm can charge the battery with 4.2 V constant voltage. 

The constant voltage charge is compared with constant current/constant voltage charge according to the simulation result. Both the constant voltage charge and the constant current/constant voltage charge can use preceding stage solar energy output electric energy to charge battery effectively; however, the defect in constant voltage charge is that the internal impedance of battery decreases gradually when the battery is charged up to a certain voltage, so that the charging current decreases gradually and the charging time is prolonged relatively. The constant current/constant voltage charge has solved this problem, the constant-current charge is implemented first, so that the full charge time of battery is shortened. Finally, the constant voltage charge is used, so that the battery will not be overcharged.

## 6. Experimental Result of Portable Solar Energy Charging System 

**Figure 14:**
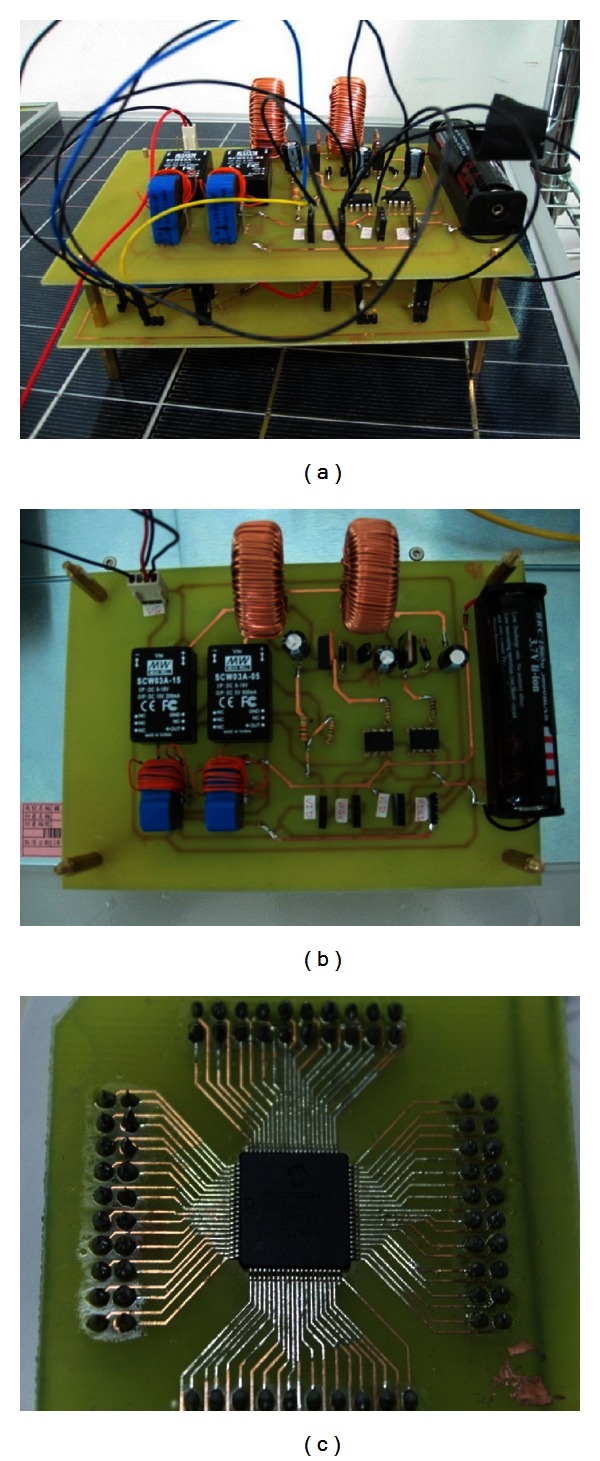
(a) hardware facility, (b) charging circuit, and (c) control chip of portable system.

For the implementation of controller, this study used the microcontrol chip PIC18F8720 developed by MicroChip to complete the overall portable design.  Figure 14(a) shows the overall portable solar energy lithium battery charging module.  Figure 14(b) shows the two-stage DC/DC converter and lithium battery used in this paper.  Figure 14(c) shows the microcontrol chip PIC18F8720 used in this paper.

**Figure 15:**
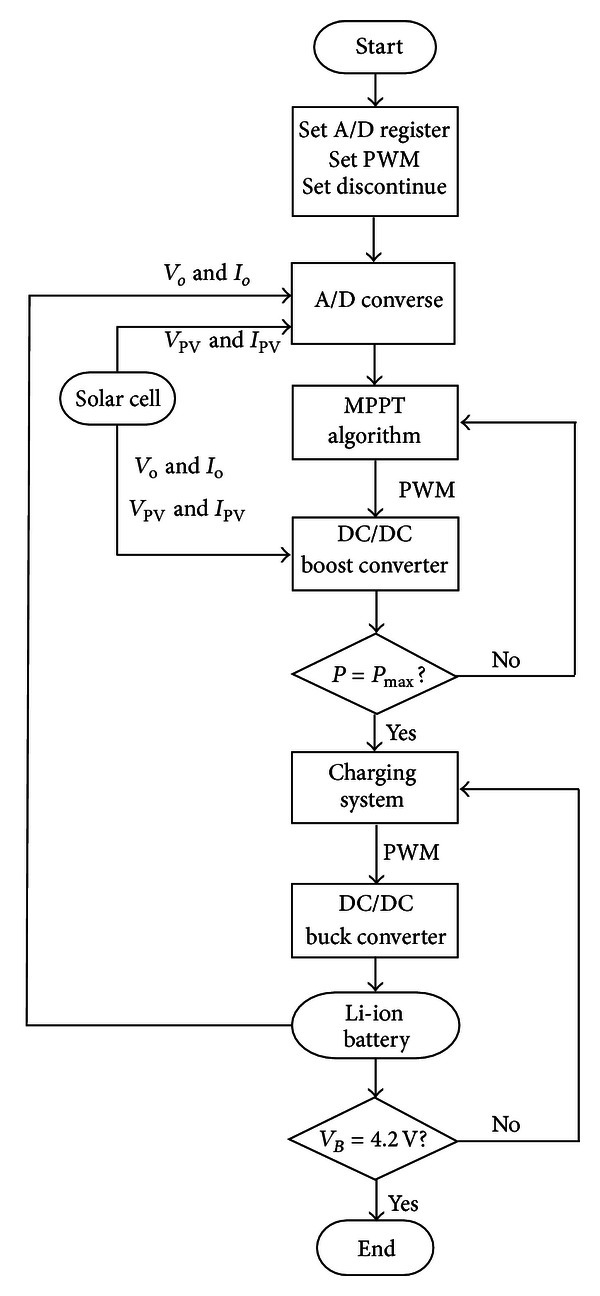
Program flow chart.

The MPPT of portable charging module and battery charging system in this study are processed by PIC18F8720. The program calculation process is shown in Figure 15.

As shown in the flow chart, the A/D buffer, PWM enable, and interrupt enable must be set at the beginning. The A/D buffer keeps reading external signal, which is the voltage and current generated by the solar cell and the voltage and current of lithium battery. First, the voltage and current values of solar cell generate PWM signal through MPPT algorithm to the DC/DC boost converter. The MPPT algorithm keeps identifying whether the solar cell has tracked maximum power point. In addition, the voltage and current values of lithium battery generate PWM signal via charge control method to the DC/DC buck converter. The charge control keeps identifying whether the battery has been charged under 4.2 V stable voltage.

### 6.1. Experimental Result of MPPT

The experimental result of MPPT algorithm is compared with simulation result. The results of different methods are simulated in standard test conditions (1 KW/m^2^, A.M. 1.5, 25°C) and changed irradiance condition (1 KW/m^2^ and 800 W/m^2^). 

The portable charging module in this study was independent PV system power supply, and the precondition was that the input voltage must be high enough to drive the microcontroller and the driver of DC/DC converter. Thus, two solar cells of the specifications used in this study were connected in series for experiment. The solar cells connected in series could double the voltage and maintain the current. In this series connection, the maximum power point voltage generated by solar energy was 9.7 V, the current was 0.72 A, and the power was 6.98 W when the irradiance was 1 KW/m^2^. The maximum power point voltage generated by solar energy was 9 V, the current was 0.66 A, and the power was 6 W when the irradiance was 800 W/m^2^. 

**Figure 16:**
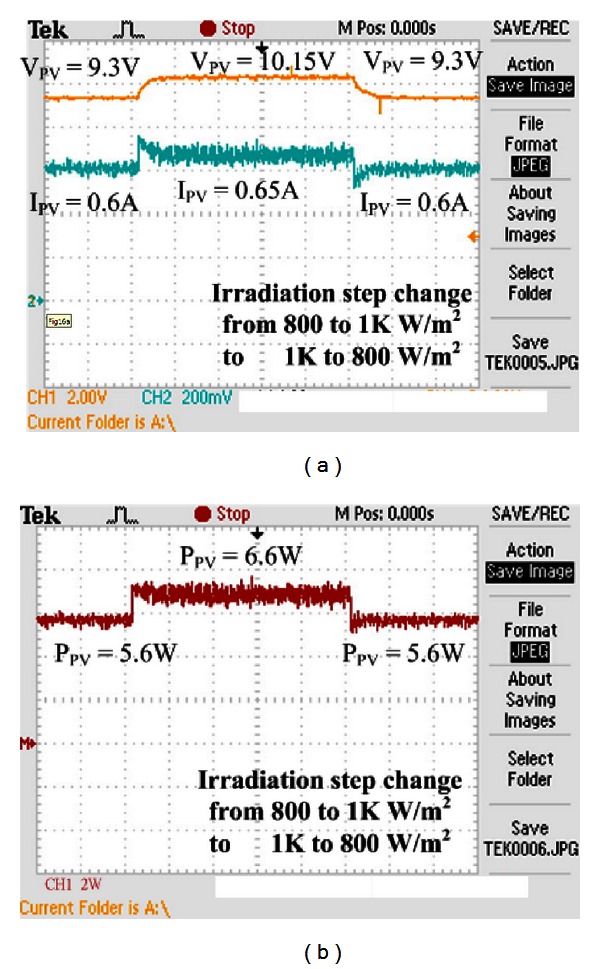
Experimental results of incremental conductance method: (a) voltage, current, (b) power when irradiance is changed (800 W/m^2^-1 KW/m^2^-800 W/m^2^) (step size is 0.005).

**Figure 17:**
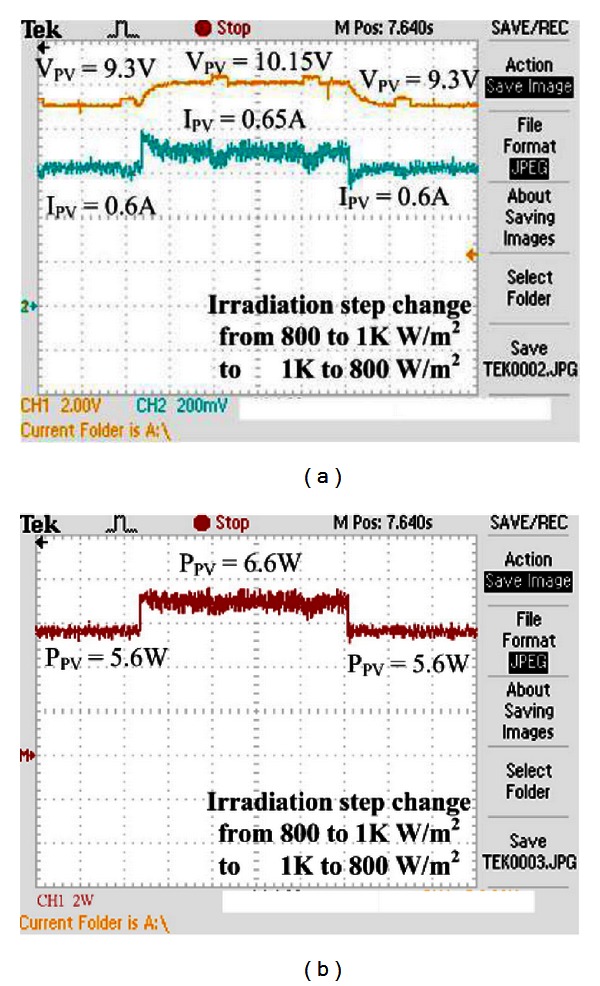
Experimental results of incremental conductance method: (a) voltage, current, (b) power when irradiance is changed (800 W/m^2^-1 KW/m^2^-800 W/m^2^) (step size is 0.01).

Figures 16 and 17 show the experimental results of voltage, current, and power of VSINC when the illumination is changed. As seen, the VSINC can make the system track the maximum power when the illumination is changed. However, according to the comparison between incremental conductance methods of step 0.005 and step 0.01, large step size incremental conductance results in very large oscillation of tracking maximum power. This oscillation causes slight power loss of the system. When the step is changed, the incremental conductance method still cannot track the maximum power point. The tracked maximum power is 5.6 W when the irradiance is 800 W/m^2^, and the tracked maximum power is 6.6 W when the irradiance is 1 KW/m^2^.

**Figure 18:**
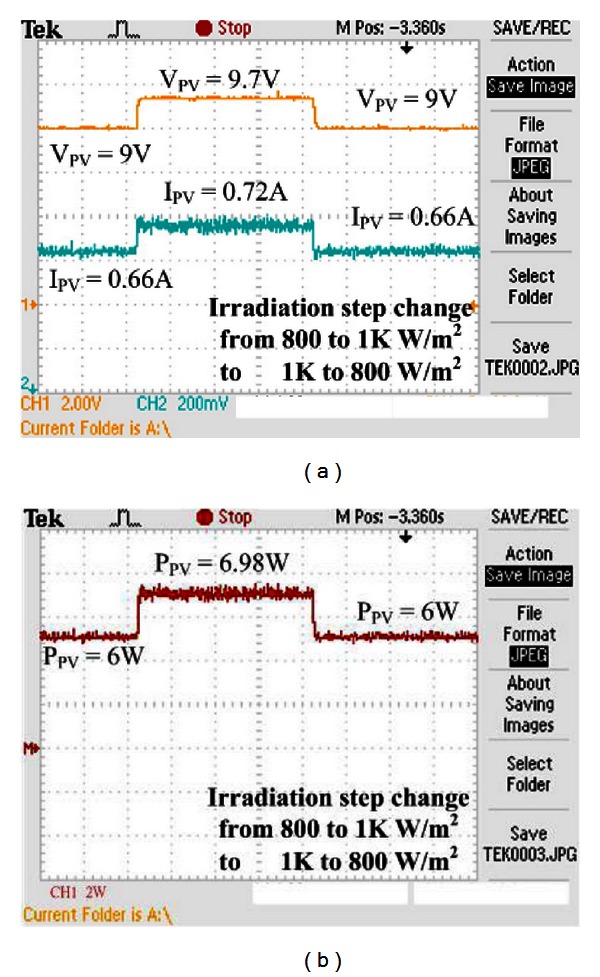
Experimental result of VSINC: (a) voltage, current, (b) power when irradiance is changed (800 W/m^2^-1 KW/m^2^-800 W/m^2^).

Figure 18 shows the experimental result of VSINC used in this paper. The yellow line in Figure 18(a) is the maximum power point voltage, and the blue line is maximum power point current. As seen, the VSINC method used in this study can implement the tracking efficiency completely in microcontroller, so that the PV system obtains maximum efficiency. It can also track the maximum power point when the illumination is changed. The system tracked maximum power is 6 W when the irradiance is 800 *W/m* ^2^, and the tracked maximum power is 6.98 W when the irradiance is 1 KW/m^2^. 

According to this experimental result, as compared with general incremental conductance method, the VSINC method has the advantages of large step size and small step size, implementing rapid tracking of maximum power point and minimum range of oscillation in simulation analysis and tracking the maximum power point in different irradiance conditions effectively. Thus, the PV system can exert maximum service efficiency for load. The VSINC method used in this study also can track the maximum power in different illuminations rapidly and has small steady-state oscillation.

The experimental results of maximum power are compared in Table 1. As seen, for fixed step size incremental conductance method, whether it is small step size (step size 0.005) or large step size (step size 0.01), the solar cell is unable to supply electric energy for the load effectively. The tracked maximum power point is 6.6 W when the step size is 0.005, the steady-state error of maximum power is 0.062 W, and the efficiency of this result is 93.6%; the tracked maximum power point is 6.6 W when the step size is 0.01, the steady-state error of maximum power is 0.132 W, and the efficiency of this result is 92.7%. The VSINC method can improve the steady-state error greatly and track the maximum power point. The overall efficiency is 98.3%, so that the solar cell can supply electric energy for the load effectively.

### 6.2. Experimental Result of the Charging System

The optimal charge control design proposed in this study was validated by battery charge experiment and compared with simulation analysis result. In the standard test conditions, the electric energy generated by the first-stage PV system MPPT passed through the second-stage buck converter before charging the lithium battery. The parameter value of PI controller of microcontroller was substituted in computer simulated optimization algorithm to search for PI value. The computer simulation revealed that the *k*_*P*_ and *k*_*I*_ values searched by PSO are better than the search results of GA; thus, this study used the *k*_*P*_ and *k*_*I*_ values searched by PSO for experiment directly to shorten charging time.

#### 6.2.1. Constant Voltage Charge

**Figure 19:**
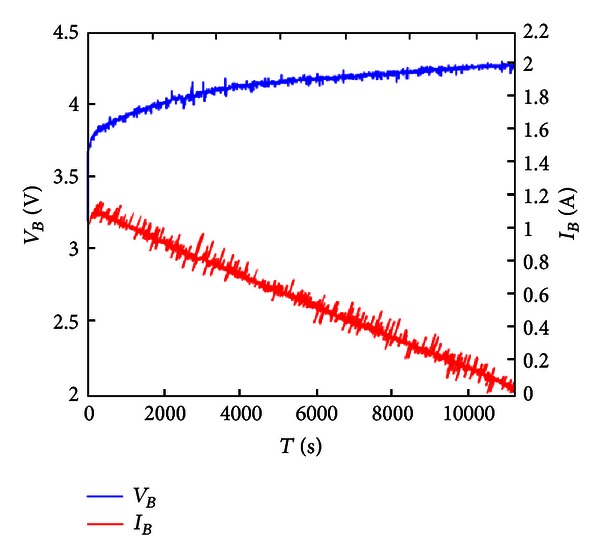
Constant voltage charge.

Figure 19 shows the experiment of battery charging voltage and current of constant voltage charge. As seen, the second-stage DC/DC buck converter used in this study can use the PI controller of microcontroller to charge the battery under constant voltage, and the charging time is about 3 h.

#### 6.2.2. Constant Current/Constant Voltage Charge

**Figure 20:**
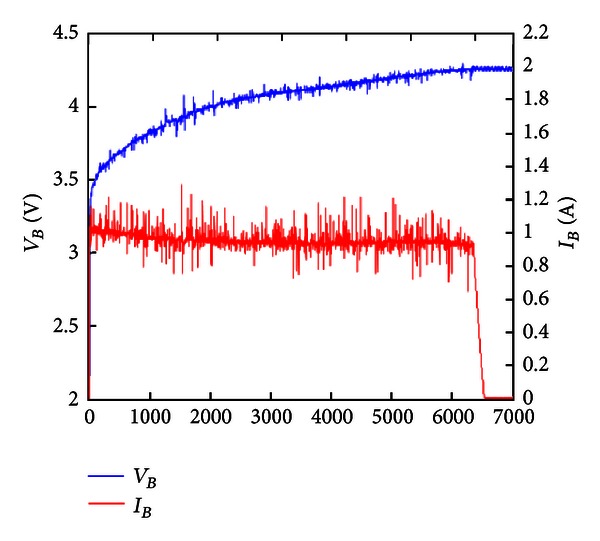
Constant current/constant voltage charge.

Figure 20 shows the experiment of battery charging voltage and current by constant current/constant voltage charge. When the battery voltage is 4.18 V, the constant current is switched to constant voltage charge. The second-stage DC/DC buck converter can use the PI controller of microcontroller to implement constant current/constant voltage charge of battery, and the charging time is about 2 h.

The experiments of constant voltage and constant current/constant voltage charge can validate the simulation results. These two methods can use the first-stage solar energy effectively. The regulation of feedback PI controller of the second-stage system stabilizes the output electric energy of the first-stage MPPT system and attains the effect of constant voltage, current, and charge. The charging time of constant current/constant voltage is much shorter than that of constant voltage charge.

## 7. Conclusions 

This study found that the proposed microcontroller 18F8720 of the portable solar cell charging module can make the first-stage DC/DC boost converter track the maximum power point of solar cell and supply electric energy for the second-stage DC/DC buck converter, so as to increase the efficiency of charging module. The main contributions of this study are listed as follows.

(1) This study proved that in the first-stage MPPT, the VSINC method can track the maximum power point faster than the fixed step size incremental conductance method and can track the maximum power point promptly when the solar irradiance is changed.
(2) The computer simulation proved that in the second-stage battery charge PI controller design, the PSO algorithm can search for the optimum controller gain value faster than GA algorithm, and its IAE value is smaller. This result means that the search efficiency of PSO algorithm is better than that of GA.
(3) According to the simulation results, the constant current/constant voltage charge can charge battery faster than simple constant voltage charge under optimal PI control, and the full charge time of battery is shorter.
(4) The experimental results proved that the two-stage system integrated with MPPT and battery charge proposed in this study can remedy the defects in the switchover control system using single-buck converter and control the MPPT of solar energy and battery charge simultaneously. This can ensure that the first-stage boost converter of system enables the system to track the maximum power point rapidly by the VSINC method, so as to stabilize the voltage. In the second-stage PI controlled charge, the gain value obtained by PSO algorithm enables the constant current/constant voltage charge to charge faster than simple constant voltage charge, and the overall charging system can implement constant voltage charge effectively.

## Figures and Tables

**Table 1:** Comparison of efficiency of MPPT algorithm.

	Maximum power point	Tracked maximum power point	Average steady-state error	Efficiency
Incremental conductance method (Step size is 0.005)	6.98 W	6.6 W	0.062 W	93.6%
Incremental conductance method (Step size is 0.01)	6.98 W	6.6 W	0.132 W	92.7%
VSINC	6.98 W	6.98 W	0.0578 W	99.1%
